# Mortal Dangers of Adult-Onset IgA Vasculitis

**DOI:** 10.7759/cureus.43624

**Published:** 2023-08-17

**Authors:** Abrahim N Razzak, Parsia Vazirnia, Shunya Hishinuma, Mohan S Dhariwal

**Affiliations:** 1 School of Medicine, Medical College of Wisconsin, Milwaukee, USA; 2 Internal Medicine, Tokyo Women’s Medical University Yachiyo Medical Center, Chiba, JPN; 3 Internal Medicine, Medical College of Wisconsin, Milwaukee, USA

**Keywords:** gastrointestinal perforation, acute kidney injury care, leukocytoclastic vasculitis (lcv), henoch-schönlein purpura (iga vasculitis), adult iga vasculitis

## Abstract

IgA vasculitis (IgAV), previously known as Henoch Schoenlein purpura (HSP), is a leukocytoclastic vasculitis subtype predominantly amongst the pediatric patient population involving IgA dominant immune complex deposits attacking small vessel walls. While it oftentimes follows upper respiratory infections and presents with palpable purpuras, IgAV can also present in the adult patient population and lead to systemic inflammation. In this case report, we present the case of an adult-onset IgAV complicated via gastrointestinal perforation, acute kidney injury secondary to IgA nephritis, and circulatory shock. A review of prognosis, complicating factors, and treatment methods was also conducted for reported adult-onset IgAV with an aim to elucidate similarities and differences to pediatric-onset IgAV. While there is no unified treatment approach, glucocorticoids and immunosuppressors such as rituximab have been observed to be an effective protocol.

## Introduction

IgA vasculitis (IgAV) is a rare subtype of leukocytoclastic vasculitis involving IgA-1 dominant systemic immune complex deposits attacking smaller vessel walls, as defined by the International Chapel Hill Consensus Conference (CHCC) [1]. IgAV is mainly predominant among the pediatric patient population and its occurrence in adults is rare [1]. Currently, the treatment options for adult-onset cases are controversial ranging from different protocols of glucocorticoids and immunosuppressants. Here, we report the case of a 66-year-old female with IgA vasculitis, whose hospital course was complicated with gastrointestinal bleeding and died due to hypovolemic, cardiovascular, and septic shock.

This case was previously presented at the Society of Hospital Medicine Wisconsin State Chapter Meeting on October 26, 2022.

## Case presentation

A 66-year-old female without any significant past medical history presented to the emergency department with new left upper extremity pitting edema, pharyngitis, odynophagia, lower extremity macular rash, and positional dizziness. Physical examination demonstrated abdominal tenderness and bilateral lower extremity petechial pinpoint lesions. She was subsequently admitted to the hospital and the initial labs showed high anion-gap metabolic acidosis, acute kidney injury (AKI), hyponatremia, elevated C-reactive protein (CRP), and high Anti-streptolysin O (ASO) titer (Table 1). Autoimmune/infectious workup, cardiac imaging, and electrocardiogram were negative; per her medical history, esophagogastroduodenoscopy was conducted which showed active duodenitis and esophagitis with ulceration. A punch biopsy from the patient's heel was performed, which confirmed direct immunofluorescence features of IgA vasculitis (IgAV) with staining; weak deposits of IgG, IgM, C3, and fibrin were also noted in blood vessels of the papillary dermis and upper dermis. As a majority of the patient’s symptoms were resolved with supportive treatment, she was discharged 13 days after the initial presentation. The patient was prescribed pantoprazole 40mg two times a day, sucralfate (Carafate) four times a day with meals, ondansetron (Zofran) 4mg three times a day with meals, and a gastrointestinal follow-up visit scheduled. 

**Table 1:** Abnormal lab results upon initial hospital admission presentation

Lab Type	Patient Values	Reference Range
Sodium Level	131mmol/L	136-145mmol/L
Chloride Level	92mmol/L	96-105mmol/L
CO2, Total	13mmol/L	22-29mmol/L
Anion Gap	26mmol/L	7-15mmol/L
Lactic Acid	3.4mmol/L	0.5-2.0mmol/L
Magnesium	1.1mg/dL	1.6-2.6mg/dL
Creatinine	1.17mg/dL	0.50-1.10mg/dL
Effective Glomerular Filtration Rate	46mL/min/1.73sqm	>=60mL/min/1.73sqm
Red Blood Cell	3.5*10^6/uL	3.7-5.2*10^6/uL
Mean Corpuscular Volume	101fl	79-98fl
Anti Streptolysin O	523.0IU/mL	<=250.0IU/mL
C-Reactive Protein	11.12mg/dL	<0.50mg/dL

**Figure 1:**
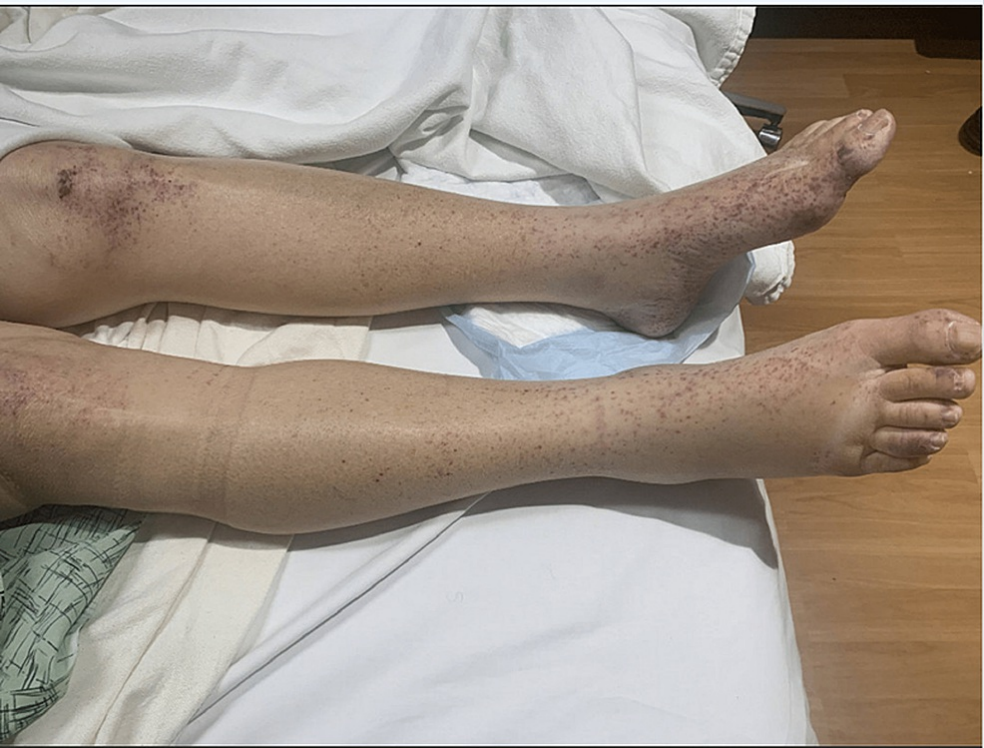
Bilateral lower extremities with non-blanching petechiae

However, the patient presented a few weeks later to the hospital with a chief complaint of generalized weakness, bloody stool, and exacerbation of her rashes. Physical examination was remarkable for increased non-blanching petechiae on knees, finger joints, feet, calves, and flanks as well as trace bibasilar crackles (Figure 1). Further labs noted anemia and bacteriuria (Table 2). The patient was subsequently readmitted due to her symptom exacerbation. She was initially started on intravenous solumedrol and rituximab.

**Table 2:** Abnormal lab results upon second hospital admission presentation

Lab Type	Patient Values	Reference Range
Sodium Level	135mmol/L	136-145mmol/L
Potassium Level	3.2mmol/L	3.4-5.1mmol/L
CO2, Total	19mmol/L	22-29mmol/L
Creatinine	1.43mg/dL	0.50-1.10mg/dL
Effective Glomerular Filtration Rate	37mL/min/1.73sqm	>=60mL/min/1.73sqm
Hemoglobin	8.0g/dL	11.5-15.5g/dL
Hematocrit	23.5%	35.0-46.0%
Prothrombin Time	13.4 seconds	9.7-13.0 seconds
Partial Thromboplastin Time	51.8 seconds	22.8-36.4 seconds
Urine Bacteria	Present	None Seen
Urine WBC	26-50/HPF	0-2/HPF
Magnesium	1.1mg/dL	1.6-2.6mg/dL
C-Reactive Protein	5.40mg/dL	<0.50mg/dL

**Figure 2:**
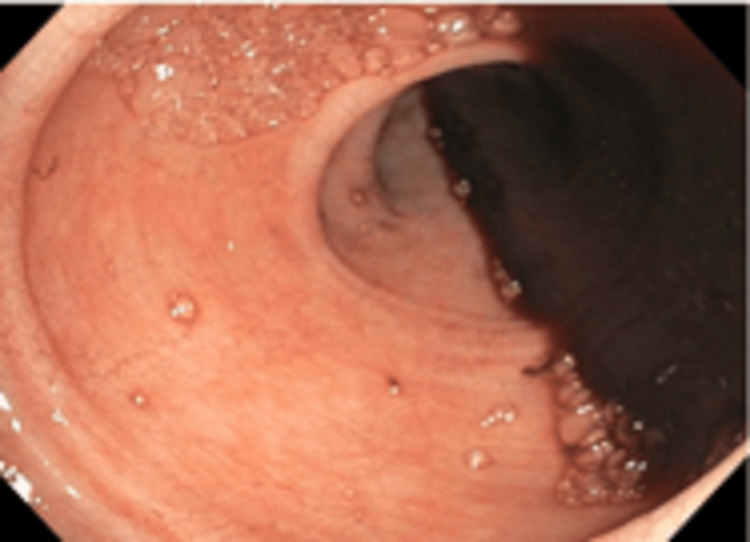
Colonoscopy finding of extensive gastrointestinal bleed

Upon her stay, the patient was evaluated by both rheumatology and nephrology to assess her acute kidney injury in which serum creatinine worsened from 1.43 mg/dL to 1.64 mg/dL. Her baseline value was near 0.7 mg/dL before clinical presentation. Given the findings of minimal proteinuria with red blood cell casts in a setting of leukocytoclastic vasculitis, nephrology confirmed her acute kidney injury was related to IgA nephritis (IgAN). The patient was also evaluated by gastroenterology due to her history of bloody stool. Subsequent esophagogastroduodenoscopy, colonoscopy, interventional radiology angiogram, and computed tomography angiography were performed which demonstrated multiple bleed locations from duodenal and esophageal ulcerations, non-bleeding jejunal ulcers, abnormal blush distal to the small bowel, and an active mid-distal small bowel bleed with intraluminal hemorrhage requiring multiple transfusions (Figure 2). Interventional radiology was consulted, but it was not amenable to intervention at that time. Her hemoglobin level at presentation was 8.0g/dL and subsequent levels taken daily fluctuated between 6.5g/dL (in which rapid response teams were called) to 8.7g/dL. Per this, she was managed with as-needed blood transfusions and started on high-dose corticosteroids in an attempt to quit the luminal manifestations of her IgAV; she was also started on rituximab. At this point, she experienced a syncopal bout with resulting hypotension.

She was then transferred to the medical intensive care unit for further management. Afterward, she was found to be in multifactorial circulatory shock secondary to septic, hypovolemic, and cardiogenic shock. Blood cultures were obtained and positive for Klebsiella; broad-spectrum antibiotics, antifungals, and vasoactive agents were initiated. The patient’s condition continued to deteriorate and was offered exploratory laparotomy and esophagogastroduodenoscopy. However, her significant other opted for transition to comfort measures during this time, and she passed away shortly after.

## Discussion

IgAV is a rare subtype of leukocytoclastic vasculitis involving IgA-dominant systemic immune complex deposits attacking smaller vessel walls, hypothetically triggered by exposure to certain antigens along with a genetic background. Two major models of IgAV have been proposed to explain the pathogenesis of the nephritis and systemic phenotypes respectively: (1) A “four-hit” theory similar to IgAN since both IgAN and IgAV nephritis are characterized by hematuria, proteinuria, and glomerular mesangial immune complex deposition; (2) Systemic effect of neutrophil activation on smaller vessels [1]. 

IgAV is mainly a pediatric disease, with around 10-20 cases per 100,000 with a peak incidence around ages of four to six years, and 90% of cases occurring under the age of 10; long-term complications and relapses are rare, with favorable outcome seen in 95% of the pediatric presentation [2]. However, on the adult side, the incidence is only around 0.8-5.1 cases per 100,000 with increased frequency in the fifth and sixth decades of life, and the mortality is increased in older patients with IgAV [2]. The most common presentation of adult-onset IgAV consists of symmetric palpable purpura, often with localized subcutaneous edema, and the diagnosis is made by biopsies typically revealing leukocytoclastic vasculitis primarily affecting the small superficial vessels. Despite the rare occurrence of adult-onset IgAV, its gastrointestinal involvement has been reported in 37-65% of IgAV cases in adults [2]. The most common symptoms include colicky abdominal pain, hematochezia, diarrhea, nausea, and vomiting. This is consistent with this case's clinical presentation as well. However, severe gastrointestinal involvement and its mortality are reported to be rare, and most commonly due to mesenteric ischemia or sepsis-related bowel perforation [2]. 

We herein reported an adult-onset case of IgA vasculitis, manifesting as a typical rash and subsequent life-threatening gastrointestinal bleeding. As discussed above, the literature has been focused on the pediatric side of the patient population because of its rare occurrence in adults. As such, there is a lack of evidence regarding the treatment options for adult-onset cases. Currently, the treatment options for adult-onset cases are controversial; corticosteroids and immunosuppressive agents such as cyclophosphamide, azathioprine, mycophenolate, cyclosporine, and rituximab have been thought to be effective [3,4]. In our case, the patient was started on high-dose corticosteroids and rituximab, but unfortunately, she passed away due to gastrointestinal bleeding complications demonstrating the lethality of IgAV.

Adult-onset IgAV cases were also reviewed in the literature; however, treatment patterns have been diverse with the utilization of corticosteroids as a main starting framework. For example, recently, there have been reports on post-COVID-19 viral infections and their relations to IgAV. Two adult-onset post-COVID IgAV cases reported similar petechial lesions and palpable purpura presentations; however, they both survived with the usage of corticosteroids [5, 6]. On the basis of treatment, one case utilized further azathioprine whereas the other used intravenous immunoglobulin and plasma exchange [5, 6]. One multi-center randomized trial demonstrated that the addition of cyclophosphamide to corticosteroids did not improve disease remission nor active visceral involvement when compared to corticosteroids alone for treatment [7]. Another observational study observed rituximab as an effective and safe therapeutic option for adult-onset IgAV due to its higher remission rates but also higher relapse rates [4]. Compared to the pediatric-onset counterpart for this pathology, it is also important to highlight the more significant prognosis for those with adult-onset IgAV given symptom reports ranging from gastrointestinal bleeding, and nephritis, to ischemia [2]. As such, there may be a greater indication for interventional autoimmune treatment protocols like monoclonal antibodies, corticosteroids, and alkylating agents.

## Conclusions

IgAV, previously called Henoch-Schoenlein Purpura, is a rare leukocytoclastic vasculitis that is commonly seen in the pediatric patient population but can also be observed in the adult patient population. They are typically diagnosed by biopsies and indicated for immunosuppressive treatment. Patients may present with significant complications and lethality such as this case with gastrointestinal perforation and shock. The treatment protocol for such cases is still up for debate given that the number of reported adult cases is very few. Further efforts to recognize adult onset IgAV are important for a quicker diagnosis and treatment.
